# Genetic and Epigenetic Mechanisms Underlying Vascular Smooth Muscle Cell Phenotypic Modulation in Abdominal Aortic Aneurysm

**DOI:** 10.3390/ijms21176334

**Published:** 2020-08-31

**Authors:** Rijan Gurung, Andrew Mark Choong, Chin Cheng Woo, Roger Foo, Vitaly Sorokin

**Affiliations:** 1 Cardiovascular Research Institute, Yong Loo Lin School of Medicine, National University of Singapore, 1E Kent Ridge Road, NUHS Tower Block, Level 9, Singapore 119228, Singapore; mdcrg@nus.edu.sg (R.G.); roger.foo@nus.edu.sg (R.F.); 2 Genome Institute of Singapore, A*STAR, 60 Biopolis Street, Genome, Singapore 138672, Singapore; 3 Department of Surgery, Yong Loo Lin School of Medicine, National University of Singapore, 1E Kent Ridge Road, NUHS Tower Block, Level 8, Singapore 119228, Singapore; andrew_choong@nuhs.edu.sg (A.M.C.); surwoocc@nus.edu.sg (C.C.W.); 4 Department of Cardiac, Thoracic and Vascular Surgery, National University Hospital, National University Health System, 1E Kent Ridge Road, NUHS Tower Block, Level 9, Singapore 119228, Singapore

**Keywords:** abdominal aortic aneurysm, vascular smooth muscle cells, single nucleotide polymorphism, epigenetics, extracellular matrix, phenotypic switching, miRNA, lncRNA, histone acetylation, DNA methylation

## Abstract

Abdominal aortic aneurysm (AAA) refers to the localized dilatation of the infra-renal aorta, in which the diameter exceeds 3.0 cm. Loss of vascular smooth muscle cells, degradation of the extracellular matrix (ECM), vascular inflammation, and oxidative stress are hallmarks of AAA pathogenesis and contribute to the progressive thinning of the media and adventitia of the aortic wall. With increasing AAA diameter, and left untreated, aortic rupture ensues with high mortality. Collective evidence of recent genetic and epigenetic studies has shown that phenotypic modulation of smooth muscle cells (SMCs) towards dedifferentiation and proliferative state, which associate with the ECM remodeling of the vascular wall and accompanied with increased cell senescence and inflammation, is seen in in vitro and in vivo models of the disease. This review critically analyses existing publications on the genetic and epigenetic mechanisms implicated in the complex role of SMCs within the aortic wall in AAA formation and reflects the importance of SMCs plasticity in AAA formation. Although evidence from the wide variety of mouse models is convincing, how this knowledge is applied to human biology needs to be addressed urgently leveraging modern in vitro and in vivo experimental technology.

## 1. Introduction

Abdominal aortic aneurysm (AAA) is identified as the dilatation of the infra-renal region of the abdominal aorta. It is characterized by >3.0 cm increase in aortic diameter or ≥50% of normal diameter and commonly presented as fusiform-shaped. The prevalence of AAA varies across different population and race with a range of 1.3–5% according to screening programs in the UK and US [1]. The major epidemiological risk factors related to AAA include male gender, age of more than 65 years old, and a history of smoking [2,3]. Other risk factors include atherosclerotic disease, hypertension, ethnicity, and family history of AAA. The AAA shares many risk factors with atherosclerosis but maintains its unique pathological characteristics. Pre-aneurysmal and aneurysmal infra-renal aortic dimension have been reported to correlate with non-AAA related cardiovascular mortality [4]. However, unlike atherosclerosis, AAA susceptibility and progression are negatively associated with diabetes mellitus [5]. However, a correlation between AAA and these conditions are not always positive and linear. Particularly in type II diabetic patients, the risk of developing AAA is reduced by approximately 50% [6].

AAA pathogenesis is a complex process characterized by aortic wall remodeling that leads to its weakening. In summary, it could be divided but not limited to: (1) Alteration of connective tissue protein and their architecture; (2) connective tissue degradation due to the imbalance between matrix metallopeptidases (MMPs) and tissue inhibitor of metallopeptidases (TIMPs); (3) chronic inflammation and cytokine release; (4) vascular smooth muscle cell (VSMC) trans-differentiation and cell death [7]. Moreover, compelling evidence shows that VSMCs are involved in all the above-mentioned pathophysiological changes that take place in the AAA wall. Furthermore, VSMCs in the differentiated state have the remarkable ability to acquire different phenotypes, including synthetic, fibroblast, and macrophage-like phenotypes, which occurs in the aortic wall of AAA patients [8].

Another reason to believe that VSMCs play an important role in the AAA lies in the field of embryology. AAA formation in the specific region of the abdominal aorta correlates with differential embryological development of the descending aorta. One of the main differences during the development of the descending and abdominal aorta is VSMCs origin. VSMCs in coronary arteries are well known to be derived from the epicardium, those in the ascending aorta and arch vessels are derived from the neural crest, and those in the descending aorta are derived from mesodermal somatic precursors layer. In adulthood, though the abdominal aorta presents three distinct layers like other aortic regions, there are histological and genetic differences across the different VSMCs lineages [8,9].

Although AAA is a well-investigated condition, an available therapy that can tackle constant aneurismal growth is currently lacking. From a clinical perspective, a greater understanding of the genetic and epigenetic mechanisms in depth would allow improved medical therapy to stabilize aortic wall, which could lead to the prevention of disease progression. This would, in turn, also help avoid devastating complications such as aortic rupture. There have been a few drug classes that have gone into clinical trials such as doxycycline, angiotensin-converting enzyme (ACE) inhibitors, and a few studies evaluating statins. However, conclusive evidence suggesting effective reduction in size or growth rate has yet to be reported, though some trials are still ongoing [10,11,12]. Moreover, therapeutic advancement would positively affect surgery and stenting application [13]. Thus, we have presented this review with the aim of providing a comprehensive overview of genetic and epigenetic discoveries pertaining to VSMCs underpinning AAA pathobiology.

## 2. Genetics of AAA

AAA has shown to cluster within certain families and there is evidence showing strong genetic component in AAA risk. Twin studies have reported that heritability could be as high as 70% [14]. The Swedish Twin registry reported that a monozygotic twin has a 24% probability of having an aneurysm as opposed to only 4.8% in dizygotic twins if the other twin has an aneurysm. Another large twin study in Denmark reported that the phenotypic variance determined by genetics is estimated to be 70–80%, whilst non-shared environment effects (such as infections, smoking, or occupational exposure) contributes to 20–30% [14,15]. Positive family history was shown to approximately double the risk of developing AAA [16]. In line with this, population studies have shown that ~19% of AAA patients reported a first-degree relative with the condition as well, considerably higher than 2–10% reported by unaffected controls [17,18,19]. 

This genetic inheritance appears to be autosomal in nature, though it could present as either dominant or recessive [20]. This highlights the importance of distinguishing familial cases from sporadic AAA when studying genetic risk factors as they could be different genetic entities. There has been evidence showing that familial AAA cases were also more prone to having a ruptured AAA and less likely to have heart disease compared to sporadic cases [21]. A Dutch study reported that although familial AAA cases shared a similar cardiovascular risk profile with sporadic AAA cases, the former group was shown to be more at risk of poorer outcomes after endovascular repair with higher requirement of repeated procedure after primary intervention [22].

The heritability of AAA can be classified as either having a Mendelian, single-gene cause, or having a non-Mendelian form with a more complex cause due to a variation of genes and downstream molecular pathways. Rare genetic diseases such as Ehlers-Danlos syndrome type IV, Marfan syndrome, Loeys-Dietz syndrome, and fibromuscular dysplasia, which can cause abdominal aortic aneurysms, are examples of Mendelian heritability [23]. However, the majority of AAA patients do not have these diseases as epidemiological analyses have revealed that only 10–20% patients with AAA have at least one relative with genetic disease [21]. This could suggest that a more complex mechanism, rather than single-gene variance may underlie AAA heritability. There is more compiling evidence suggesting that non-Mendelian heritability related to epigenetic and downstream translational mechanisms play a major role in vascular disease and VSMC plasticity related to that process [24].

Observations of AAA co-occurring with thoracic aortic aneurysm (TAA) in patients have led to studies focusing on shared genetic risk between dilating arterial conditions such as AAA, TAA, and intracranial aneurysm [25,26]. Several loci such as 9p21 and 15q21 were reported to contribute to potential pan-aneurysmal effects, though substantial genetic overlap between the three conditions was lacking [25].

## 3. VSMC Development and AAA

In the early stages of vasculogenesis, the recruitment and differentiation of precursor VSMCs is driven by chemoattractants like platelet-derived growth factor-β (PDGFβ) and transforming growth factor-β (TGFβ) and the formation of a network of tracks made of endothelial cells (ECs), leading to vasculature maturation in a highly regulated process. [27]. Vascular maturation involves the activation of many genes and their associated signaling molecules including those encoding sonic hedgehog (Shh), Notch, Ephrin, Tie, and Angiotensin (Ang) in specific locations across the vascular system including the descending and abdominal aorta. VSMC recruitment and differentiation in the descending aorta (dorsal aorta at relevant embryological stage) is associated with the presence of the notch ligand Jagged 1 (Jag1) in ECs and leads to a unique induction of VSMCs through Notch2 and Notch3 activation. The process of VSMC recruitment and media formation in the descending aorta at the latter stages of vasculogenesis leads to the replacement of mesodermal (lateral plate)-derived SM22*α*+ cells by somite-derived VSMCs [28].

The unique embryological origin of VSMCs in abdominal aorta leads to a specific landscape of the genetic transcript in these cells. The infra-renal abdominal area is different in cellular content, genetic activity, and histological structure, which render it more susceptible to specific clinical conditions [29,30]. Of note, elastin degradation in the infra-renal aorta is one of the most potent mechanisms that promote the dilation of this specific aortic segment. There are also differences in elastin content between the aortic arch and descending aorta due to primitive SMCs of the aortic arch being replaced by VSMCs migrated from the neural crest, which are better withstand the higher pulse pressure and ejection volume by laying down more elastic lamellae during development and vascular remodeling [31,32]. Altogether, there is growing evidence showing that genetic and epigenetic modulation in VSMCs acquired in vasculogenesis during embryological development may play a major role in the pathophysiological mechanisms involved in AAA development. The mesodermal VSMCs precursors of the abdominal aorta respond differently to various cytokines and growth factors compared to the neural crest precursor VSMCs [33]. Human lateral plate derived mesodermal VSMCs uniquely respond to the administration of cytokines like interleukin-1β (Il-1β), leading to MMP9 and TIMP1 upregulation, which causes ECM degradation [34]. Homocysteine, an amino acid containing sulfur is reported to be involved in elastolysis, collagen deposition, and aortic compliance was found to stimulate neural crest vascular SMC proliferation and promote synthetic activity but did not cause any effects on mesoderm-derived SMCs [35]. Transforming growth factor β (TGFβ), a mediator of cell growth and differentiation, which is able to induce DNA synthesis and collagen production in neural crest-derived VSMCs, does not have a similar effect on mesodermal-derived VSMCs [36]. These differences between neural crest-derived VSMC and somite-derived VSMCs have been confirmed in in vivo experiments. Continual infusion of the vasoactive peptide angiotensin II, associated in the previous experiments with vascular remodeling and atherosclerosis, lead to the development of aneurysms in the suprarenal abdominal aorta in apolipoprotein E knock out (*ApoE*-/-) mice without affecting the thoracic aorta [37]. In the ascending aorta and arch, angiotensin II response by neural crest-derived VSMCs promotes hyperplasia. A distinctly different response by mesoderm-derived VSMC was observed, marked by increased activity of the transcriptional regulator inhibitor of differentiation 3 (ID3), a mediator of mitogenic signaling [38]. 

## 4. VSMC-Mediated Extracellular Matrix Production and Degradation in AAA

The ECM, synthesized and processed by fibroblast-like VSMCs, plays a valuable role to maintain the blood containing function of the arterial wall. For instance, the enzyme lysyl oxidase is synthesized and secreted by VSMCs and is crucial for maturation of fibrillar structures such as elastin and collagen, rendering them insoluble. The ECM is comprised of microfibrillar structures such as elastin and collagen, fibronectin, and fibrillin. In healthy vessels, the elastin functions to resist vasodilation, whereas collagen functions to resist rupture [39]. Multiple studies have shown that VSMCs not only produce ECM but also involved in release and maturation of MMPs and TIMPs, which, in turn, control integrity and degradation of extracellular fibrillar structure of aortic wall. Thus, MMP/TIMP production balance plays an important role in the formation of AAA [40,41]. Furthermore, the proteolytic and oxidative environment in the aortic wall can lead to the partial disappearance of VSMCs seen in AAA. Plasmin and elastase, in particular, have been shown to cause cell detachment and death [42,43].

### 4.1. MMPs in AAA

**Figure 1:**
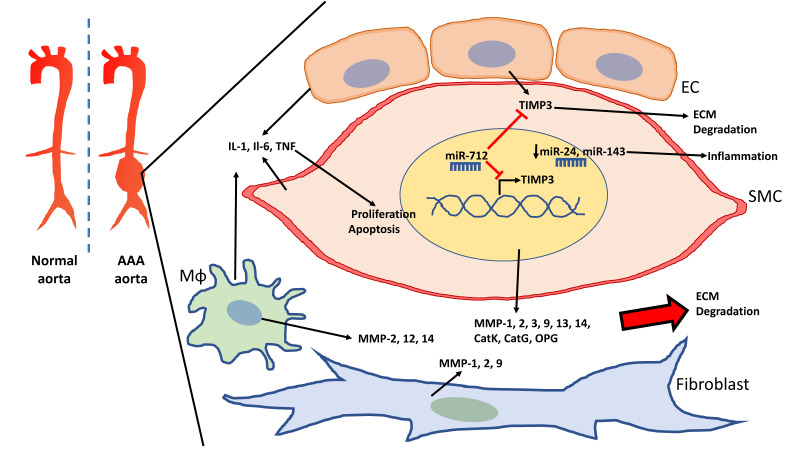
Schematic of the abdominal aortic aneurysm (AAA) aorta and vascular cells involved in the degradation of the extracellular matrix (ECM). Vascular smooth muscle cells (VSMCs), endothelial cells (EC), and macrophages (Mɸ) release cytokines that elevate inflammation in AAA and promote VSMC proliferation as well as apoptosis. VSMCs, macrophages, and fibroblasts also contribute to the elevation of matrix metallopeptidases (MMPs), which lead to ECM degradation. miR-712 targets tissue inhibitor of metallopeptidase (TIMP)3 to further promote ECM degradation in AAA. Downregulation of miR-24 and miR-143 lead to increased inflammation.

MMPs play a major role in the progression of both AAA and TAA. In the normal aorta, MMPs are produced in VSMCs, ECs, and adventitial fibroblasts [44]. In AAA, they can also be released by inflammatory cells such as neutrophils and macrophages. VSMCs are also able to produce a wide variety of MMPs elevated in AAA, including MMP-1, -2, -9, -13, and -14, which lead to degradation of the aortic wall (Figure 1). As a result, many genome-wide association studies (GWAS) have studied single nucleotide polymorphisms (SNPs) in specific MMP genes to investigate their association with AAA development.

The MMP family of endopeptidases is wide-ranging and consists of members that target specific components of the ECM. Each member has five domain structures: a signal domain, a prodomain, hinge region, a hemopexin domain, and a catalytic site [45]. MMPs are classified into 6 groups based on their substrate specificity: Collagenases, gelatinases, stromelysins, matrilysins, membrane-type MMPs, and other MMPs (Table 1).

### 4.2. Other Mediators of ECM Degradation: Cathepsins, Osteoprotegerin and Kallikreins

Regulators of protease and MMP activities that cause VSMC apoptosis and tunica media degradation in AAA have also been reported. Cathepsin K (CatK) and CatG are both highly expressed in vascular tissues in AAAs and can activate MMPs, collagenase, and elastase. CatK is expressed by ECs and osteoclasts; CatG is expressed by SMCs, neutrophils, and mast cells. Both CatK and CatG deficiency in AAA mouse models showed reduced VSMC apoptosis, elastin degradation, and lesion formation [61,62]. Osteoprotegerin is another driver of protease and MMP activity that is also positively associated with human AAA growth.

Other proteases produced in vascular tissue include kallikreins. They aid in the releasing of kinins (bradykinin and lysyl-bradykinin), which in turn cause the eicosanoids, nitric oxide (NO), and endotheliu- derived hyperpolarizing factor (EDHF) release. These processes induce vasodilatation, natriuretic and diuretic effects. Lowering of kallikrein levels has led to remodeling of the arterial wall [63]. A SNP (rs5516) in the *KLK1* promoter was reported to be enriched in AAA patients compared to controls and in large AAAs compared to small AAAs [64].

### 4.3. TIMPs

The condition of the ECM and aortic wall is dependent on the balance between metallopeptidases and their inhibitors, TIMPs. Insufficient activity or low expression of TIMPs could promote ECM degradation and VSMC modulation. This is seen in the aneurysmal aortic wall as decreased mRNA levels of TIMPs have been reported [65,66]. TIMP1-deficient mice were shown to develop larger aneurysms after porcine pancreatic elastase (PPE) perfusion compared to wild-type mice [67].

Tilson et al. conducted the first DNA sequencing analysis of *TIMP1* in six AAA patients and revealed a silent polymorphism in two of the patients [68]. Another study reported a SNP (C>T) at nucleotide position 434 and showed an association between the C-allele and female AAA patients [69], though the patient cohort was reportedly small (*n* = 20). A subsequent sequence analysis conducted in 50 patients for the *TIMP1* gene failed to detect any mutations associated with AAA [70].

TIMP2 level decreased in AAA patient serum and the ratio of *MMP2/TIMP2* mRNA is elevated in AAA tissue compared to control aortic tissue [48,71]. A DNA sequence analysis of the *TIMP2* gene revealed a SNP at nt 573G>A, with the G allele being significantly more frequent in male AAA patients (*n* = 64) compared to male controls (*n* = 29) [69]. However, a similar analysis of the TIMP2 gene failed to find this association [72]. The role of TIMP2 is rather complex as it also plays a role in MMP2 activation by functioning as a co-activator of proMMP-2 [73,74]. In mice, *Timp2* knockout resulted in protection against CaCl_2_-induced AAA [75].

TIMP3 level is elevated in AAA and may be attributed to its protective role in disease. TIMP3 deficiency was shown to trigger the AAA in AngII-infused mice. The knockout of *Timp3* gene led to the adverse remodeling of the abdominal aorta, reduced collagen and elastin proteins, and elevated proteolytic activities, which was not seen in wild-type mice [76]. However, no association between TIMP3 and AAA were identified. 

The role of TIMP4 in AAA has been least investigated, though increased expression was shown in hyperhomocysteinemia-associated aortic aneurysm in human and mice [77].

### 4.4. miRs Regulating ECM Degradation

MiRNAs are short (~20 base pair long), single-stranded non-coding RNA that directly regulate gene expression and exert a wide range of cellular functions [78]. They negatively regulate gene expression by degrading mRNA or mimicking small interfering RNA (siRNA) and inhibiting translation. miRNAs are initially transcribed as pi-miRNAs, longer primary transcripts, that are processed by the ribonuclease enzyme complex Drosha/DGCR8, followed by Dicer, and incorporation into the RNA-induced silencing complex (RISC) [79]. Mature miRNA is able to pair with a highly conserved seed sequence within the 3’ untranslated region (UTR) of potentially multiple target genes [78]. A wide variety of miRNAs have been studied in AAA that target specific genes to regulate VSMC plasticity as well as ECM degradation and inflammation (Table 2).

#### 4.4.1. miR-29b

In both the PPE and AngII infusion models of *ApoE*-/- mice, AAA development was associated with decreased aortic miR-29b expression [80]. AAA was also associated with increased expression of *Col1a1, Col3a1, Col5a1, and Eln* genes. Overexpression of miRNA-29b increased AAA expansion and aortic rupture rate, while miRNA-29b inhibition using anti-miR-29b decreased AAA expansion and promoted aortic fibrosis. Both human aortic VSMCs and human fibroblasts express miR-29b, though treatment with transforming growth β (TGFβ) significantly decreased miR-29b expression only in aortic fibroblasts, suggesting that these cells primarily mediate profibrotic effects. Similar to the murine models, human AAA patients displayed downregulated miR29b and upregulated *COL1A1, COL3A1, COL5A1*, and *ELN* in aneurysmal tissue [80]. In contrast, miR29b was increased in early aneurysm development in the Marfan syndrome mouse model [81]. This was associated with increased apoptosis presented by elevated cleaved caspase-3 and -9, enhanced caspase-3 activity, and decreased expression of antiapoptotic proteins—myeloid cell leukemia 1 (Mcl-1) and B cell lymphoma 2 (Bcl-2). Interestingly, miR-29 activity was repressed by nuclear factor kappa-light-chain-enhancer of activated B cells (NFκB) signaling.

#### 4.4.2. miR-205

miR-712 in mice and its human/murine homolog miR-205 have been shown to suppress TIMP activity in response to AngII-induced enhanced aortic MMP activity and promote AAA formation [82]. Both miR-712 and miR-205 stimulated MMP activity by direct targeting of TIMP3 and reversion inducing cysteine-rich protein with kazal motifs (RECK) in ECs of AngII-infused *ApoE*-/- mice. The effects of miR-712 were also observed in VSMCs though the effect of miR-205 on VSMCs was not reported. The upregulation of four AngII-sensitive miRNAs, miR-205, -21, -133, and -378, identified in the mouse model, was also observed in aortic samples of AAA patients (Figure 1).

### 4.5. HDAC Inhibitors Regulating MMPs-2 and -9

Histone deacetylases (HDACs) are potent epigenetic regulators of gene transcription. They comprise of 18 molecules divided into four classes: I, II (comprising IIa and IIb), III, and IV. Class I and IIa HDACs have been shown to modulate the expression of VSMC genes involved in AAA and regulate differentiation, contractility, proliferation, inflammation, and ECM deposition [83]. HDACs and lysine histone acetylase transfers (HATs, also known as KATs), play key roles in post-translational modification as they can attach or remove an acetyl group at the lysine residue. Acetylation neutralizes their positive charge of histone tails and results in the loosening of the histones and associated DNA. This loose chromatin structure allows accessibility for transcription factors and promotes transcriptional activation. In converse, deacetylation leads to a more compact chromatin structure [84]. 

Galan et al. reported increased expression of HDACs 1 and 2 (from class I) and HDACs 4 and 7 (both from class IIa) in aortic samples of AAA patients compared to the control group [85]. High expression levels of the four HDACs were particularly seen in the tunica media of aneurysmal samples comprised of VSMCs and inflammatory cells. A similar HDAC expression profile was also observed in aortic samples from angII-infused *ApoE*-/- AAA mice.

#### 4.5.1. MS-275 and MC-1568

The class 1 HDAC inhibitor MS-275 and class IIa inhibitor MC-1568 decreased AAA expansion and improved survival in mice [85]. Both inhibitors were able to reduce systemic inflammatory response such as macrophage and lymphocyte infiltration and elastin and collagen fiber disorganization. Additionally, both MS-275 and MC-1568 inhibited the upregulation of pro-inflammatory markers and MMP-2 and MMP-9 activity observed in the mouse AAA model (Figure 1).

#### 4.5.2. Metacept (MCT)

**Figure 2:**
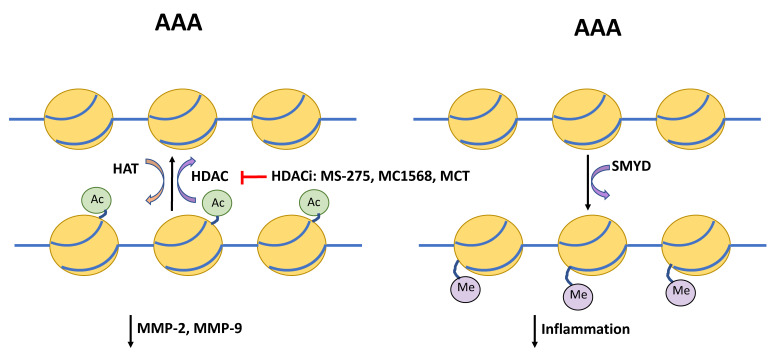
Schematic depicting regulators of histone acetylation and DNA methylation in AAA. Histone deacetylases (HDAC) and histone acetylase transferases (HAT) provide post-transcriptional modification via removing or attaching an acetyl group at the lysine residue. HDAC inhibitors (HDACi) MS-275, MC1568, and metacept (MCT) inhibit acetylation, which has been shown to reduce levels of matrix metallopeptidases (MMP)-2 and-9, and in turn protect the extracellular matrix from degradation. The SET and MYND domain-containing (SMYD) promoter region is hypomethylated in AAA compared to controls. SMYD itself is able to methylation of promoters of interleukin-6 (IL-6), tumor necrosis factor α (TNFα), and hypoxia inducible factor 1 α (HIF1α) and suppress inflammatory signaling.

Similarly, another HDAC inhibitor, metacept-1 (MCT-1), was also shown to reduce AAA incidence in AngII infused *ApoE*-/- mice [86]. MCT also reduced expression of MMP2 in human VSMCs in vitro and MMP-2 and MMP-9 in AAA mouse tissue samples (Figure 2).

#### 4.5.3. HATs in AAA

The activity of myocardin, a cardiac- and muscle-specific coactivator of the transcription factor serum response factor (SRF), was shown to be enhanced by p300, a HAT that associates with the activation domain of myocardin [83]. Class II HDACs have shown to interact with myocardin in separate binding domains and suppress myocardin-induced smooth muscle differentiation. 

A study comparing AAA and healthy aortic samples reported a wide variety of HATs that were significantly higher in disease [87]. KAT2B, KAT3A, KAT3B, and KAT6B were among the highest expressed in AAA tissue. KAT6A expression was shown to correlate with the contractile VSMC marker myosin heavy chain 11 (MYH11) as well as inflammatory CD3+ and vascular cell adhesion molecule 1 (VCAM-1)+ ECs. Thus, KAT expression profiles may potentially serve as biomarkers of AAA and risk of rupture but also shed light on the complexity of the balance between HAT and HDAC activity in AAA.

## 5. Smooth Muscle Cells and Inflammation in AAA

Overall, inflammation is a large component of AAA though the origin of immune cells and the inflammatory cascade has been a much-debated subject [44,88]. There is evidence showing that macrophages have a hemopoietic and tissue-specific origin. More recently, lineage tracing techniques have confirmed that VSMCs are also able to transdifferentiate into macrophage-like cells.

Phenotypic switching of VSMCs to macrophage-like LGALS3+ and CD68+ cells has been shown in human AAA lesion samples. Lineage tracing in the *ApoE*-/- mouse model showed that phenotypically transdifferentiated VSMCs comprised of ~30% of the cell population within the lesion of the brachiocephalic [34]. Furthermore, phenotype modulation could be assessed through differential expression of key genes such as *MYH11*, *alpha-smooth muscle* actin *(ACTA2)*, calponin 1 *(CNN1)*, and transgelin (*TAGLN)* in VSMCs.

The study using in situ hybridization and proximity ligation assays reported that the dimethylation of histone H3 Lysine K4 (H3K4me2) of the *MYH11* locus was specific to the smooth muscle cells in human and mouse sections. Remarkably, this epigenetic mark was retained in the cells in phenotypic modulated cells within atherosclerotic lesions, suggesting the potential robustness of certain epigenetic markers in SMCs [24].

### 5.1. Upregulated Cytokines in AAA

In general, inflammation is a hallmark AAA formation of aneurysms and its role in AAA is well supported compared to the limited studies shown in TAA. Neutrophils infiltrate to the aortic site very early, though only transiently, and are sources of proteases, MMPs, and reactive oxygen species and can start degradation of the ECM and aortic wall. However, most of the studies on neutrophil involvement include animal experimental work.

The function of smooth muscle is heavily influenced by macrophage activation and infiltration in AAA as these inflammatory cells are responsible for a lot of the production of MMPs, cytokines, chemokines, and ability to remove cellular debris [44]. Macrophages can also release netrin-1, a protein known to promote macrophage retention in tissues, to drive AAA pathogenesis. *Netrin-1* deletion resulted in an MMP3-dependent reduction in AAA formation [89].

Upregulation of the cytokines such as interleukin 1α (IL1α), IL1β, IL-6, and tumor necrosis factor α (TNFα) are seen in AAA. Of interest, the very same cytokines are produced by VSMCs [90,91,92]. IL1α, IL1β, and TNFα, which are also produced by macrophages and are potent mitogens of VSMCs, implying that the cytokine in the aortic wall may be produced via autocrine regulation [86,93,94,95,96]. IL1β and TNFα in combination with interferon γ promote VSMC apoptosis [97]. It is also known that IL-1β plasma levels in patients with AAA and coronary artery disease (CAD) are significantly higher than in CAD patients without AAA. IL-1β likely promotes apoptosis via MMP upregulation and activation in patients with CAD and AAA [98,99,100,101,102,103]. Cytokine elevation in AAA patients is well documented, though their precise mechanisms are a matter of great debate. Six SNPs in the *IL1A*, *IL1B*, and *IL1RN* genes have been investigated in a study but no associations with AAA were found [104]. Two small case-control studies and a meta-analysis failed to find an association between a SNP in the *IL1B* gene (nt3953 C>T) and AAA [104,105,106].

TNFα can induce VSMC apoptosis by causing the increase of caspase-3 activity and inhibiting gap junctions, specifically connexin 43 [107]. The A-allele of a SNP located at nt-308 has been shown to cause a six- to nine-fold increase in *TNFα* mRNA in B-cells compared to the G-allele, though the association between the A-allele and AAA was not seen [105,108].

IL-6 is predominantly expressed in leukocytes such as macrophages and T-cells and though they are not normally expressed by VSMCs, they can be expressed by aortic VSMCs under conditions of elevated aortic wall tension, as would be the case in AAA [92]. A large case-control study analyzed data identified a single SNP (rs1800796; -572 G>C) in the *IL-6* promoter as an independent risk factor for AAA, though it was only present in 1.5% of the cases [109]. Other SNPs were detected in subsequent studies but showed no association [105,110].

TGF-β is the most extensively studied cytokine in TAA, though it may play a role in AAA mechanistically [111,112]. In contrast to how TGF-β signaling drives TAA formation, systemic blockade of TGF-β activity or genetic deletion of its downstream signaling protein SMAD3, a transcriptional modulator and member of TGF-β receptor regulated SMAD family, worsened AngII and CaCl_2_-induced AAA in mice [113,114]. Other study showed that the peptide antagonized thrombospondin-1 could promote AAA in AngII-infused *ApoE* deficient mice through inhibition of TGF-β1-mediated Smad2/3 signaling [115]. A study analyzing data from large Australian and New Zealand cohorts examined for 49 SNPs in the *TGFBR2* gene and reported one SNP (rs1078985) that showed weak association with AAA, though it did not hold up to multiple testing [116]. Two more SNPs in the *TGFRB2* gene (rs1036095 and rs4522809) reported in a large Dutch study also showed association with AAA [117].

### 5.2. Inflammation-Associated Genes

The SET and MYND domain-containing 2 (*SMYD2*) is a highly expressed gene in muscle cells that is involved in myofibril organization. It interacts with three functional groups of molecules: (1) chromatin remodeling and histone modification, (2) DNA replication, and (3) repair and molecular chaperones. It methylates heat shock protein 90 (Hsp90) and improves sarcomere stability [118]. The *SMYD* promoter region of SMCs is significantly hypomethylated in AAAs compared to controls. In line with this, Hsp90 inhibition also reduces AAA formation in murine models [119]. *SMYD2* methylation in aortic SMCs correlated with gene expression, with cytoplasmic abundance in smooth muscle in the tunica media being lower in AAA compared to non-aneurysmal controls. Moreover, *SMYD2* can methylate the promoters of *IL-6* and *TNFα* gene promoters, leading to the inhibition of NFkB and extracellular signal-regulated kinase (ERK) signaling pathways [120]. Thus, hypomethylation of *SMYD* may result in decreased vascular structural stability and increased inflammation, promoting the AAA phenotype (Figure 2).

There are several SNPs on the 1p13.3 locus that have been identified by several AAA meta-GWAS studies located near the *PSRC1*, as well as other genes including *CELSR2* and *SORT1* [121,122]. Expression quantitative trait loci and chromatin interaction data showed that *SORT1* gene may be most likely to be influenced by the SNPs [123]. *SORT1* was first seen expressed in the liver regulating plasma LDL-cholesterol through interactions with promoter of pro-protein convertase subtilisin/kexin type 9 (*PCSK9*) acidic sphingomyelinase and apoB_100_ in hepatocytes [124]. In 2018, Zhang et al. reported that it is also expressed in VSMCs and could induce VSMC calcification in vitro and in rats [125].

### 5.3. Reduced Contractile Phenotype/Anti-Inflammatory Inducing miRNA in AAA

#### 5.3.1. miR-24

Downregulation of the miR-23b-24-27b cluster in murine AAA models was reportedly associated with AAA in the PPE-infusion mouse model, in which miR-24 showed the most significant inverse correlation of its predicted targets in array profiling studies [126]. In both the PPE and AngII *ApoE* AAA mouse models, miR-24 was shown to target chitinase 3-like 1 (*Chi3l1*), an inflammation marker of AAA disease progression. This limited inflammation by blocking IL-8 and monocyte chemoattractant protein 1 (MCP1/CCL2) production by VSMCs and M1 subtype macrophages and inhibiting new macrophage recruitment and survival. Decreased plasma miR-24 expression was observed in AAA patients as well as in the murine AAA models. Furthermore, IL-6 was shown to decrease miR-24 expression via NFkB-mediated pathway in siRNA-mediated knockdown models of the *RelA* and *NFkb1* genes. *CHI3L1* was another gene that showed the ability to promote aortic VSMC migration in vitro via c-Jun N-terminal kinase (JNK) and ERK phosphorylation [127].

#### 5.3.2. miR-143/145

The miR-143/145 cluster highly expressed in VSMCs and has been shown to be most abundantly expressed in the heart and aorta [128]. The miRNAs miR-143 and miR-145 are known to induce a differentiated, contractile phenotype in smooth muscle cells and are also shown to be downregulated in aneurysms compared to control aorta [128]. Their expression in blood is significantly lower in coronary artery disease (CAD) patients compared to healthy individuals [129]. The studies implicate that it may be involved in modulating the pro-inflammatory, MMP-producing VSMC phenotype to stabilize AAA [130].

## 6. Smooth Muscle Cell Plasticity and Apoptosis in AAA

VSMCs in the aneurysmal wall have impaired contractility and greater susceptibility to apoptosis and senescence [11,131]. In addition, a variety of genetic and epigenetic regulators have been reported to modulate the VSMCs toward either a dedifferentiated synthetic (proliferative) phenotype typically seen in disease or a senescent phenotype. In vitro and animal studies have shown that targeting these regulators could lead to a deeper understanding of AAA pathophysiology and advancements in therapeutics.

### 6.1. Genetic and Epigenetic Mechanisms Promoting the Synthetic SMC Phenotype

#### 6.1.1. Cyclin-Dependent Kinase 2B Antisense (CDKN2BAS)

**Figure 3:**
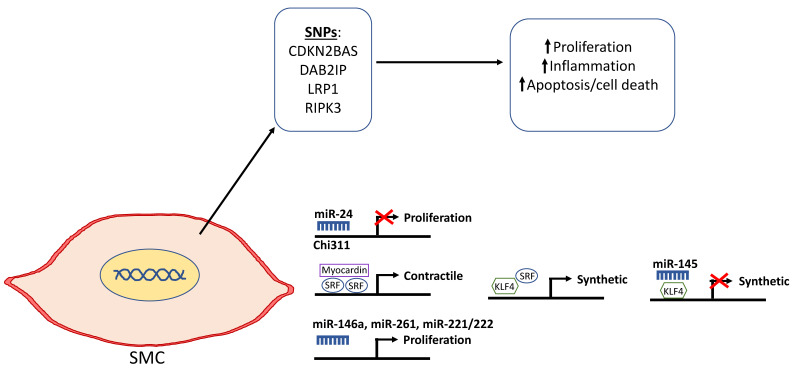
Schematic depicting the genetic and epigenetic mechanisms that are involved in the phenotypic modulation of smooth muscle cells (SMCs) in AAA. Single nucleotide polymorphisms in selected genes including cyclin dependent kinase 2B antisense (CDKN2BAS/ANRIL), DAB2IP, lipoprotein receptor-related protein 1 (LRP1), and receptor-interacting serine/threonine-protein kinase 3 (RIPK3) have been shown to be associated with the diseased SMC phenotype as depicted by increased proliferation that leads to vascular remodeling, inflammation, and cell death. miR-24 selectively targets Chi311 to inhibit proliferation. Kruppel-like factor 4 (KLF4) normally represses myocardin to downregulate SMC differentiation and promote the synthetic phenotype. miR-145 can repress KLF4 and, in turn, inhibit de-differentiation into the synthetic SMC phenotype. miR-146a, miR-261, and mir-221/222 have been reported to promote SMC proliferation.

There have been a number of alleles discovered through GWAS in the last two decades that directly or indirectly affect smooth muscle proliferation and apoptosis. SNPs at the 9p21.3 locus have been consistently associated with AAA. The non-coding RNA gene *CDKN2BAS*, also known as antisense non-coding RNA in the INK4 locus (ANRIL), encodes a lncRNA regulating the expression of cyclin-dependent kinase inhibitor 2B associated with AAA development and is located within the p15/CDKN2B-p16/CDKN2A-p14 alternate reading frame (ARF) gene cluster [26,132,133,134]. PPE-infused *Cdkn2b*-/- mice developed larger aortic aneurysms compared to wild-type mice. The *Cdkn2b*-/- mice contained fewer VSMCs that exhibited increased apoptosis in the aortic wall [135]. *Cdkn2b* knockdown in the smooth muscle cells in vitro led to increased growth arrest and apoptosis. Furthermore, immune-histochemical analysis of human tissue samples showed that *CDKN2B* was mainly expressed in VSMCs and reduced levels of *CDKN2B* in AAA aortic tissues samples were observed compared to controls. When a 70 kbp region, including the SNP (rs10757278) and portions of the *Cdkn2bas* gene but not the two closest protein genes *Cdkn2a* and *Cdkn2b*, was deleted in mice, cardiac and vascular *Cdkn2a* and *Cdkn2b* expressions were reduced. The rate of proliferation of SMCs isolated from aorta of these mice was double of those from same strain controls [136]. Thus, patients with the risk allele at the 9p21 locus have lower *CDKN2B* expression levels, which in turn enhances apoptosis, causing thinning of the medial layer of the aortic wall and ultimately making it more prone to dilation seen in AAA (Figure 3) [137]. 

#### 6.1.2. Disabled Homologue 2 Interacting Protein (DAB2IP)

Another AAA GWAS studied assessed approximately 300,000 SNPs and located 3 SNPs on chromosome 9p21 associated with AAA at genome-wide significance and 22 other SNPs that were also significantly associated. One SNP (rs7025486) that had a highly significant P-value was within intron 1 of *DAB2* interacting protein, also known as apoptosis signal-regulating kinase 1 (ASK1) interacting protein (*AIP1*) [138]. DAB2IP is a Ras GTPase-activating protein family member that is normally highly expressed in VSMCs and ECs and has been shown to inhibit proliferation and intimal expansion. Knockout or knockdown of *DAB2IP* in VSMCs was shown to significantly enhance IFN-γ-induced JAK-signal transducer and activator of transcription proteins (STAT) signaling and IFN-γ dependent migration and proliferation of VSMCs [139]. By binding to JAK2, DAB2IP could inhibit IFN-γ signaling through STAT1/STAT3 along with their pro-survival effects (Figure 3).

#### 6.1.3. Lipoprotein Receptor-Related Protein 1 (LRP1)

A large multi-center GWAS in AAA revealed nine loci associated with AAA as well as a single SNP (rs1466535) on chromosome 12q13.3 located within intron 2 of the gene encoding low-density lipoprotein receptor-related protein 1 (*LRP1*), which showed high association with AAA [133]. This association was also confirmed by a later study [140]. Smooth muscle-specific LRP1 inactivation by crossing SM22Cre transgenic mice with *Lrp^flox^* animals has been shown to cause aneurysm, overexpression, and abnormal activation of platelet-derived growth factor (PDGF), disruption of the elastic layer, VSMC proliferation, and cholesterol-induced atherosclerosis [141]. LRP1 plays an important role in maintaining integrity of the vascular wall, which could be via Smad signaling [142]. Thus, in patients where LRP1 signaling is disrupted, its protective effects could be suppressed, leading to AAA development (Figure 3).

#### 6.1.4. Receptor-Interacting Serine/Threonine-Protein Kinase 3 (RIPK3)

Medial VSMC depletion is a hallmark characteristic in both AAA and TAA. Whether VSMC death is an active pathological event or a consequence of growth arrest or apoptosis has not been fully investigated. Cellular apoptosis and necrosis have been categorized as distinctly separate forms of cell death. More recently, studies have pointed towards a third form of death known as necroptosis, where certain types of necrosis are regulated by orchestrated signaling networks [143]. Receptor-interacting serine/threonine-protein kinase 3 (RIPK) has been reported as an important regulator of this programmed necrosis (Figure 3). *Ripk* deletion reduced VSMC necroptosis and inhibited the development of PPE-induced AAAs in C57BL/6 mice [43].

#### 6.1.5. Notch Homolog 1 Translocation-Associated (NOTCH1)

In humans, the *NOTCH1* gene encodes a transmembrane receptor expressed on many cell types including smooth muscle and ECs and is involved in development and differentiation. Notch1 signaling was reported to be activated in the AngII-infused *ApoE* knockout AAA mouse model. Aneurysm incidence could be reduced via *Notch1* haploinsufficiency and pharmacological inhibition of NOTCH1 signaling through the prevention of macrophage accumulation to the aneurysm site [144].

#### 6.1.6. HIF-1α

The transcription factor hypoxia-inducible factor-1α (HIF-1α) regulates multiple biological pathways, including stimulating cellular proliferation, angiogenesis, cell apoptosis, migration, and metabolism to adapt to low oxygen availability [145,146]. Its transcription is low in normoxia and elevated in hypoxic conditions. Its upregulation is prominent in various vascular diseases including pulmonary hypertension, atherosclerosis, and AAA [88]. HIF-1α overexpression is present in the rupture edge of AAA human tissues. VSMCs derived HIF-1α can promote vascular remodeling by upregulating cytokines and chemokines, leading to increased AngII signaling [147,148]. A study by Gabel et al. reported that genes positively associated with progression of larger AAA were also associated with angiogenesis and vascular remodeling and highly expressed in fibroblasts and VSMs of the aneurysmal wall [149]. Additionally, these genes as well as genes exclusively upregulated in ruptured AAA were functionally converged through processes mediated by the HIF-1α pathway.

### 6.2. Elevated miRNA Promoting Synthetic Phenotype

#### 6.2.1. miR-21

miR-21 has been shown to inhibit VSMC apoptosis and protect against AAA formation in *Apoe*-/- mice (Figure 3) [150]. Aortic miR-21 expression levels were elevated in both the PPE- and angII-infusion *ApoE* knockout AAA models compared to controls group. Overexpression of miR-21 through lentiviral transduction reduced phosphatase and tensin homolog (*Pten*) expression and led to increased phosphorylation and activation of AKT, which regulates a number of kinases, transcription factors, and regulatory molecules, promoting a proliferative and antiapoptotic VSMC phenotype. Furthermore, the miR-21 upregulation was augmented by nicotine, further implicating the role of miR-21 as a driver of vascular remodeling in smoking, a major modifiable risk factor for AAA.

#### 6.2.2. miR-146a

miR-146a was also reported to be elevated in AAA and promote VSMC proliferation and survival and involved in AAA pathogenesis (Figure 3) [151]. Sun and colleagues reported that miR-146a targeted the 3’ untranslated region (UTR) of the Kruppel-like factor 4 (*KLF4*) transcription factor and caused VSMC proliferation in vitro and vascular neo intimal hyperplasia in balloon-injured rat carotid arteries. *KLF4* has an anti-proliferative effect on VSMCs through upregulation of p21 and differentiation genes SM22α and α-smooth muscle actin and downregulation of the dedifferentiation gene *SMEMB*. Inhibiting miR-146a using anti-miR transfection in VSMCs increased *KLF4* expression, while miR-146a overexpression reduced *KLF4* levels.

#### 6.2.3. miR-26a

miR-26a promotes VSMC proliferation while inhibiting cellular differentiation and apoptosis and altered TGF-β signaling (Figure 3) [152]. In human aortic SMCs, inhibition of miR-26a via anti-miR transfection accelerated VSMC differentiation, promoted H_2_O_2_-induced apoptosis, and inhibited proliferation and migration. Conversely, its overexpression blunted differentiation. miR-26a inhibition led to increased SMAD1 and SMAD4 expression, while overexpression inhibited SMAD-1, suggesting that miR-26a promotes proliferation via altering TGFβ signaling. This would affirm past studies that showed that TGFβ signaling through SMAD-1/5/8 induces VSMC differentiation and inhibits proliferation [153]. Zhang et al. reported that SMAD4 or TGFβ receptor II deficiency in VSMCs led to aortic aneurysm formation in mice and increased cathepsin S and MMP-12 [154]. miR-26a was downregulated in both the PPE and AngII *ApoE*-/- AAA mouse models [152].

#### 6.2.4. miR-221/222

miR-221 and miR-222, which have been implicated in cancer cell proliferation, have also been shown to regulate VSMC proliferation and neo-intimal hyperplasia (Figure 3) [155]. Their expressions were identified in rat carotid arteries after angioplasty, which were upregulated and localized in VSMCs after injury to the vascular wall. They were also upregulated after PDGF and serum stimulation in VSMCs. Knockdown of miR-221 and miR-222 using antisense oligonucleotide-mediated miRNA depletion led to decreased rat aortic SMC proliferation in vitro via activation of p27 and p57, which are negative regulators of VSMC proliferation. miR-221 was also crucial for PDGF-induced cell proliferation by downregulating p27 and c-Kit expression [156].

### 6.3. lncRNAs Associated with Pro-Proliferative SMC Upregulated Alongside Apoptosis

#### 6.3.1. H19

*H19* was one of the earliest lncRNAs identified in AAA and was upregulated in the AngII and PPE mouse models of AAA compared to controls [157]. *H19* knockdown using site-specific antisense oligonucleotides (LNA-GapmeRs) significantly inhibited aneurysm growth in both mouse models. The medial VSMCs and adventitial fibroblasts of normal non-dilated tissue expressed *H19*. AngII infusion led to *H19* upregulation in medial VSMCs of dilated aortic tissues, which was abolished through *H19*-targeted deletion by GapmeRs. AngII/H19-induced VSMC apoptosis was dependent on the expression of HIF1α, which interacted with proto-oncogene transformed mouse 3T3 cell double minute 2 (*Mdm2*), and prevented *Mdm2*-mediated reduction of p53, a factor that triggers aneurysm expansion.

#### 6.3.2. Plasmacytoma Variant Translocation 1 (PVT1)

Plasmacytoma variant translocation 1 (*PVT1*) is a lncRNA that increased expression in tissues from AAA patients and AngII-infused *ApoE*-/- mice [158]. PVT1 expression was increased by AngII in VSMCs in vitro. Furthermore, overexpression of *PVT1* in the AngII-induced AAA mouse model led to increased VSMC apoptosis, elevated MMP-2, and MMP-9, reduced TIMP-1, and differentiation into the synthetic SMC phenotype. Knockdown in vitro and silencing in vivo using shRNA lentiviral vector against RNA-*PVT1* exhibit reversed AAA-induced alterations.

### 6.4. Downregulation of Protective Genes in AAA

#### 6.4.1. Peroxisome Proliferative-Activated Receptor Gamma (PPARG)

Peroxisome proliferative-activated receptor gamma (PPARγ), a nuclear hormone receptor known for playing a critical role in glucose and lipid metabolism, is expressed in VSMCs. Its activation by thiazolidnediones leads to a contractile VSMC phenotype with reduced proliferation and oxidative stress [159]. PPARγ activation has also been shown to inhibit osteoproteregrin expression in human aortic SMCs [160]. The PPARG c.34G>C polymorphism weakly associated with AAA, while c.1347C>T was strongly associated with increased AAA growth [161]. VSMC-targeted deletion of *Pparγ* in mice has been shown to cause systemic hypotension possibly via increased β2-adrenergic receptor expression as well as pulmonary hypertension [162,163]. Interfering PPARγ signaling has also shown to induce vascular dysfunction, hypertrophy, and remodeling [164,165]. Thus, a polymorphism affecting PPARγ activation might implicate loss of protective effects on VSMCs, leading to aortic wall degradation.

#### 6.4.2. Serpin Proteinase Inhibitor B9 (SERPINB9)

Serpin proteinase inhibitors are able to protect the aortic wall and their decrease in expression levels has been implicated in AAA. Serpin proteinase inhibitor, clade A, member 1 (SERPINA1), also known as α1-antritrypsin (α1-AT), was shown to inhibit VSMC cell apoptosis and ECM degradation induced by elastase in VSMCs and mammary artery cultured ex vivo [166].

SERPINB9 was reported to inhibit apoptosis of human VSMCs [167]. A genome-wide methylation analysis in peripheral blood mononuclear cell DNA from AAA patients and control subjects identified increased methylated regions in calponin 2 (*CNN2*) and adenylate cyclase 10 pseudogene 1 (*ADCY101*). The same study also showed decreased methylation in kelch-like family member 35 (*KLHL35*) and *SERPINB9* in AAA samples compared to controls [168]. Gene expression was higher for *SERPINB9* and lower for *CNN2* in AAA patients. Immuno-histochemical analysis showed that the aortic media and intima was more prominently stained for CNN in AAA patients and SERPINB9 in control patients. CNN2 is an actin-binding protein, implicated in vascular development and shown to be upregulated in stretched vascular walls [169].

### 6.5. Reduced miRNAs Promoting Contractile Phenotype

#### miR-143/145

Both miR-143 and miR-145 induce the contractile VSMCs phenotype and their expression is significantly diminished in aneurysms compared to controls tissue [128]. They are encoded by highly conserved genes that lie in close proximity with each other on chromosome 5 in humans and chromosome 18 in murine [170]. The transcription factors serum response factor (SRF) and cardiac NK-2 transcription factor (Nkx2-5) can independently activate miR-143 and miR-145 expression and synergistically upregulate expression to promote de-differentiation of contractile VSMCs. Myocardin, a transcriptional coactivator of SRF, was shown to augment miR-143/145 expression when in combination with SRF, and cause additive effects with both SRF and Nkx2-5 [171]. The loss of miR-143 in a KO mouse model utilizing a lacZ reporter also led to decreased miR-145 expression, implicating their co-dependence [128].

Phenotypic differentiation caused by miR-143 and miR-145 has been studied in in vitro and in vivo models. Overexpression of miR-145 in VSMCs increased the gene expression of differentiation markers like smooth muscle (SM) α-actin, calponin, and SM-myosin heavy chain. It also helps to maintain a spindle-like shape and inhibits VSMC proliferation [172]. Homozygous miR-143/145 KO mice had a reduced number of contractile and increased number of synthetic VSMCs in the aorta and femoral artery compared to wild-type controls [128]. Smooth muscle layers of aorta and other arteries were remarkably thinner in miR-145 and miR-143/145 KO mice compared to wild-type or miR-143 KO mice due to a greater reduction in actin-based stress fibers, indicating the more prominent role of mir-145. Ultrastructural analyses revealed dilated endoplasmic reticulum that were increased in number in miR-143, -145, and -143/145 knockout mice, indicative of synthetically active VSMCs.

The two miRNAs can work in a myriad of pathways to induce VSMCs differentiation. miR-145 represses KLF4, which is normally able to interact with SRF, repress myocardin and calmodulin kinase II-δ, promote neointimal proliferation, and downregulate VSMC differentiation marker genes (Figure 3) [159,160,161]. Another target of miR-145 is *KLF5*, which also represses myocardin expression and is activated in AngII-induced VSMC proliferation via the angiotensin I-triggered ERK and p38 mitogen-activated protein kinase (MAPK) pathways [172]. miR-143 was reported to target Ets like gene 1 (Elk-1), which competes with myocardin to bind to SRF and inhibits VSMC differentiation [173]. miR-143 was also shown to attenuate the expression of versican, a chondroitin sulfate proteoglycan of the ECM produced by synthetic VSMCs, upon binding to myocardin [174]. Both miR-143 and miR-145 can target actin-dependent SRF coactivator myocardin-related transcription factor-B (MRTF-B) and Adducin-3 (ADD3), which are membrane skeletal proteins that bridge the membrane and actin cytoskeleton and aid in cell motility [175,176,177,178]. Thus, targeting MRTF-B and ADD3 resulted in inhibition of the migratory phenotype.

## 7. Smooth Muscle and ROS in AAA

Reactive oxygen species (ROS) plays an integral role in the development of AAA, possibly more so than in TAA, as they result in VSMC apoptosis, MMP activation, and induction of pro-inflammatory genes in AAA [44]. Increased superoxide levels were some of the first observations seen in AAA [183]. Administration of vitamin E as an antioxidant has shown to lead to decreases in AAA size and rupture in animal models [184]. Similarly, catalase overexpression in smooth muscle was shown to prevent mechanical changes in the aortic wall in the AngII model and AAA formation inhibition in the CaCl_2_ model [185]. Oxidative stress was also shown to be linked with histone acetylation in various models of cardiovascular diseases leading to downstream proinflammatory signaling [83]. Increased ROS production has been associated with molecular changes in vascular cells that lead to increased levels of cytokine release, upregulated adhesion molecules that allow leukocyte infiltration, upregulation of MMPs and other proteases, and induction of apoptotic cell death pathways (ie. Fas and perforin) that culminate towards the structural deterioration of the aortic wall in AAA [186].

### 7.1. NADPH Oxidase

A major source of ROS in AAA is NADPH oxidase (NOX), which was reported to be upregulated in AAA patient aortic tissue [183]. VSMCs as well as ECs, fibroblasts, and infiltrating leukocytes are able to form O_2_- and other oxidants such as H_2_O_2_ via NADPH oxidase. Mechanical and pulsatile stretch was shown to induce ROS production by NADHPH oxidase in VSMCs, which subsequently led to NFkB activation and *MMP*-2 expression and activation [187,188]. VSMCs and tissue infiltrating macrophages can release cytokines such as TNFα that can also activate NADPH oxidase [189,190]. Other activators of NADPH oxidase include growth factors such as AngII and platelet-derived growth factor (PDGF), lipid mediators such as leukotrienes, and lysophosphatidic acid, and oxidized low-density lipoproteins LDL [186]. Genetic deletion of the p47 subunit of NADPH oxidase has been reported to attenuate AAA in the *ApoE*-/- and AngII induced mouse models [191,192]. *Nox2* deficiency in the angII-induced AAA mouse model led to increased AAA diameter and formation through increasing vascular inflammation in the LDL receptor and decreased overall ROS production and macrophages polarization towards the anti-inflammatory phenotype [193]. Another study showed that genetic knockdown Nox1, Nox2, Nox4, or p47phox prevented development of AAA in AngII-infused mice. This observation was accompanied by improved NO and tetrabiopterin bioavailability, reduced superoxide production, and recoupled endothelial nitric oxide synthase [194]. In the same study, two novel NOX4 mutations were identified in AAA patients that were associated with elevated H_2_O_2_ levels.

### 7.2. Low Antioxidant Gene Expression in AAA

Antioxidant profile in AAA can also help determine severity of ROS in patients. Reduced levels of catalase expression are observed in animal AAA tissue, while reduced levels of glutathione peroxidase and paroxinase-1 are observed in AAA patients. Both increased and decreased levels of superoxide dismutase (SOD), which catalyze O_2_- to O_2_ and hydrogen peroxide, have been reported in patients with AAA [44,195,196]. Specific isoforms of SOD have also been reported with manganese-dependent SOD (*MnSOD*) expression increased and extracellular SOD decreased in the PPE-induced rat model [197,198].

A family of mitochondrial transporter proteins that are known to provide protection from cells against oxidative damage are uncoupling proteins (UCPs) [199,200]. *Ucp* mRNA and protein expression are upregulated in *AngII*-induced AAA of mice [201]. Knockout of the subtype *Ucp*-*2* was reported to increase AAA incidence, promote elastin degradation and destruction of the aorta via upregulation of MMP2 and MMP9, increase oxidative stress, decrease SOD, and induce VSMC apoptosis in *ApoE*-/- AAA mice compared to *Ucp*+/+ aneurysm and wild type controls enzyme system.

### 7.3. ROS and Histone Acetylation

The biological effects of oxidative stress in various cardiovascular diseases including AAA have been shown to be partly mediated by histone acetylation regulated by HAT and HDAC activities, leading to the activation of NFκB signaling [83]. Elevated HAT and decreased HDAC expression levels were associated with increased ROS in vitro studies. ROS was shown to raise HAT activity and, in turn, increase histone acetylation, promoting inflammation in several cell lines [202]. AngII-induced ROS was reported to increase acetylated cyclphilin, a pro-inflammatory agent, in VSMCs. This was associated with increased phosphorylated ERK1/2, MMP-2 activation, elevated ROS production in VSMCs as well as increased intercellular adhesion molecule 1 and VCAM-1, which cause monocyte adhesion, compared to non-acetylated cyclophilin A [203]. H_2_O_2_ also was shown to increase gene acetylation by increasing HAT and suppressing HDAC activities [202]. Interestingly, application of the HDAC inhibitor hydroxamic acid inhibited streptozotocin-induced Nox1, Nox 2, and Nox4 expression and ROS production in mouse aorta [204]. Thus, the field of histone acetylation in AAA appears complex but also rapidly expanding.

## 8. DNA Methylation in AAA

DNA methylation is a powerful epigenetic mechanism that is important in the maintenance of DNA structure, chromosome stability, X chromosome inactivation, and can regulate elements such as transposons and retrotransposons and gene expression [205]. DNA methylation occurs when a methyl group is added to a region where a cytosine base 5’ to a guanine by DNA methyltransferase enzymes forming a CpG dinucleotide [206,207]. A cluster of CpGs is referred to as an CpG island. Repression of genes via DNA methylation can occur by either preventing transcription factor binding to their binding sites through methylation of cytosine (C) or occupying the promoter region by one of the methyl-cytosine binding (MBD) proteins [208]. Though it is classically known to inhibit gene expression, increased methylation is also associated in some instances with increased gene expression, as methylation can be necessary for transcriptional binding [209,210,211].

There has been limited investigation of the role of DNA methylation in AAA, relative to other epigenetic mechanisms. A large study that assessed peripheral blood mononuclear cell DNA from AAA patients controls reported that global DNA methylation was significantly higher in men with large AAA compared to small AAA and controls [212]. VSMCs isolated from AAA patients showed altered DNA methylation levels found in four genes: v-ets avian erythroblastosis virus E26 oncogene homolog (*ERG*) with 13 hyper-methylated CpGs, IL-6 receptor (*IL-6R*) with 2 hyper-methylated CpGs, *SMYD2* with 4 hypomethylated CpGs, and *SERPINB9* with 6 hypo-methylated CpGs. There was also downregulation of *SMYD2* and *SERPINB9* in AAA, and a direct relationship between the *SMYD2* promoter methylation and expression. Downregulated *SMYD2* was observed in the AAA wall. The studies suggest that an epigenetic signature of global and CpG-specific hypermethylation of proinflammatory and pro-apoptotic genes and hypomethylation of anti-inflammatory and anti-apoptotic genes is associated with AAA formation.

A recent study showed that methylation of the SM22α gene promoter was significantly higher in AngII and CaCl_2_-induced AAA mouse tissues compared to control tissues [213]. Genetic knockdown of the SM22α in VSMCS of both models resulted in accelerated AAA formation, elevated ROS production, and NFκB activation in AAA tissues, while overexpression resulted in opposite effects. This would suggest potential association between gene-specific methylation and AAA development.

It should be noted that age, smoking, and inflammation are major risk factors of AAA that can have a substantial impact on DNA methylation patterns. Studies in aging have shown genome-wide hypo-methylation and promoter-specific hyper-methylation [214,215,216]. Cigarette smoking has shown to lower methylation levels than non-smokers. Smoking cessation resulted in partial restoration of DNA methylation status but was never completely reversed to non-smoking levels [217]; conflicting evidence showing both hypo-methylation and hyper-methylation. A genome-wide methylation study reported that inflammation and increased mortality in chronic kidney disease was associated with global DNA hypermethylation. In atherosclerosis, the underlying mechanism was reported to be based on the inactivation of suppressors of cytokine signaling (SOCS) [218]. However, it remains unclear whether DNA methylation changes are causal factors for inflammatory disease or vice versa [219].

## 9. Conclusions

The past two decades have shown tremendous growth in the understanding of molecular mechanisms underlying AAA pathobiology. Though it is not possible to include every discovery, we have attempted to report a comprehensive overview of both the genetic and epigenetic mechanisms associated with the role of smooth muscle cells in AAA formation. Though there are no curative therapies for AAA, a deeper understanding of the two areas of molecular biology and how they integrate together could help pave the way forward toward better patient care and AAA therapeutics.

## Figures and Tables

**Table 1:** Key MMPs that are synthesized by VSMCs and/or mediate ECM degradation in AAA.

	Expression	Enzyme Group	Substrate	Function
MMP-1	VSMCs, fibroblast, leukocytes	Collagenase	Collagen (I, II, III, VII, VIII, X), MMP-2, MMP-9, gelatin, proteoglycans	Released predominantly by mesenchymal cells [45,46]. Relies on presence of active MMP3 and plasmin to promote transition of proMMP-1 to active MMP-1 [46]
MMP-2	VSMCs, fibroblasts, macrophages	Gelatinase	Gelatin, collagens (I, IV, V, VII, X, XI, XIV), MMP-1, MMP-9, MMP-13, elastin, fibronectin, laminin	Degrades elastin and fibrillar collagen. Largely expressed by VSMCs [47,48]. Transition of contractile to synthetic VSMC phenotype (as seen in AAA) induces MMP-2 production and enables migratory properties [49]. Mediated by other MMPs (1, 7, membrane type MMPs) [50]
MMP-3	Fibroblasts, epithelial cells, macrophages	Stromelysin	Collagens (III, IV, V, IX, X), MMPs (1, 7, 8, 9, 13), fibronectin, gelatin, laminin	The 5A/6A polymorphism on the MMP-3 gene promoter region increased MMP-3 transcriptional activity and is an independent risk factor for AAA development [51]
MMP-9	VSMCs, fibroblasts, infiltrating macrophages	Gelatinase	Collagens (I, IV, V, VII, X, XI, XIV), elastin, fibronectin, plasminogen	Comprises the predominant elastases present in human AAA. Also exhibits collagenolytic and gelatinolytic activity [52]. Very low concentrations in cell cultures from normal aortic tissues [53]. Works in concert with MMP-2 and MMP-12 to promote aneurysmal degeneration [54,55]. *MMP9* C-1562T polymorphism significantly more common in AAA compared to PVD patients and control subjects [52]
MMP-12	Macrophages	Collagenase	Collagen IV, MMP-2, gelatin, elastin, fibronectin, casein, plasminogen, fibrinogen	Increased in human AAA and not seen in atherosclerotic or normal media tissues. Activity is localized in the tunica media [56]. Genetic inactivation or pharmacological inhibition of PI3-kinase delta increased MMP-12 expression and macrophage migration [57]
MMP-13	VSMCs	Collagenase	Collagens (I, II, III, IV, IX, X, XIV), gelatin, MMP-9	Enzymatic activity is localized to VSMCs of aneurysms. -77A/G polymorphism was an independent risk factor for AAA formation [51]. Nitric oxide-induced CD147 production led to increased MMP13 expression in PPE-induced AAA mice [58]
MMP-14	VSMCs, macrophages	Membrane type	Collagens (I, II, III), MMP-2, gelatin, casein, elastin, vitronectin, fibronectin, laminin	Prominent activator of proMMP-2 [59]. Primarily degrades collagens type I, II, and III. To a lesser degree, degrades gelatin, casein, elastin, fibronectin, vitronectin, and laminin causing degradation of the ECM in the tunica media and adventitia [45,59,60]

**Table 2:** miRNAs that are key players in the pathophysiology of AAA organized under three categories: ECM degradation inducing, contractile phenotype inducing, and synthetic phenotype inducing.

Category	miRs:	Key Targets	Expression	Function
ECM degradation inducing	miR-29b	*ELN, Col1A1, COL3A1, COL5A1, Bcl-2, Mcl-1*	Decreased in AngII and PPE perfusion-induced AAA mice and Marfan syndrome mice with aortic root aneurysm [81] Decreased in AAA samples [80]	Increased apoptosis. Activity was repressed by NFκB signaling
	miR-712/205	*TIMP3, RECK*	Increased miR-712 in AngII-induced AAA mouse SMCs and endothelial cells Increased miR-205 in AAA human samples [82]	Suppress TIMP activity in response to AngII-induced enhanced MMP activity
Contractile/anti-inflammatory phenotype inducing	miR-24	*Chi3l1, Mmp14, Stac2, Limd2, Marcksl1, Bcl2l11, Vav1, Prdm1*	Decreased in PPE and AngII-induced AAA in mice Decreased in AAA human plasma [179]	Blocked IL-8 and CCL production by VSMCs and M1 macrophages. Inhibited macrophage recruitment and survival. Expression was downregulated by IL-6 via NFκB signaling. Inhibited *CHi3l1*-induced VSMC migration
	miR-143	*Elk1, Versican*, protein kinase C-ε, PDGFR-α	Decreased in TAC and AngII ApoE-/- AAA mice Decreased in human aortic aneurysms [128,130]	VSMC proliferation and differentiation, actin remodeling, contractility, podosome formation and migration [130,171]
	miR-145	*Myocd, Klf4, Klf5*, Calmodulin kinase II-δ, Slit-Robo GTPase-activating protein 1 & 2, Fascin, Adducin-3		
	miR-143/145	*Myocd*, related transcription factor-B, Sling-shot 2, tropomyosin 4, ACE		
Synthetic phenotype inducing	miR-21	*PTEN, Bcl-2*	Increased in PPE- and angII-induced AAA in mice Increased in human AAA aortic samples [126,150]	VSMC proliferation and apoptosis via phosphorylation of AKT
	miR-26a	*Smad1, Smad4, Loxl, Inhbb, BAK1, PAK2, SULF1*	Decreased in PPE and AngII-induced AAA mouse models [152]	Increased proliferation/migration, apoptosis, cytokine production, TGF-β receptor pathway signaling
	miR-146a	*KLF4*	Increased in human AAA aortic samples [151,180]	VSMC proliferation, neointimal hyperplasia
	miR-221/-222	P27, p57, c-Kit	Increased in human AAA aortic tissue [181]	VSMC proliferation, neointimal hyperplasia [155,156,182]

## Abbreviations

AAA	Abdominal aortic aneurysm
ACTA	Actin alpha 2
ADCY101	Adenylate cyclase 10 pseudogene 1
ADD3	Adducin-3
Ang	Angiotensin
ApoB_100_	Apolipoprotein B100
Bcl-2	B cell lymphoma 2
C>T	Cytosine to Thymine single nucleotide polymorphism
CaCl_2_	Calcium chloride
CAD	Coronary artery disease
CatK/G	Cathepsin K/G
CDKN	Cyclin-dependent kinase
Chi311	Chitinase 3-like 1
CNN	Calponin
COL	Collagen
CpG	Cytosine-guanine dinucleotide cluster
DNA	Deoxyribonucleic acid
EC	Endothelial cell
ECM	Extracellular matrix
EDHF	Endothelium-derived hyperpolarising factor
ELN	Elastin
ERG	E26 oncogene homolog
ERK	Extracellular signal regulated kinase
G>C	Guanine to Cytosine single nucleotide polymorphism
H_2_O_2_	Hydrogen peroxide
H3K4me2	Dimethylation of lysin residue 4 on histone 3
HDAC	Histone deacetylases
hESC	Human embryonic stem cell
HIF1α	Hypoxia-inducible factor 1 α
Hsp90	Heat shock protein 90
ID3	Inhibitor of differentiation 3
Il-1β	Interleukin-1β
Il-6	Interleukin-6
iNOS	Inducible nitric oxide
Jag1	Jagged1
JNK	c-Jun N-terminal kinase
KAT	Lysine histone acetylase transferase
KLF	Kruppel-like factor 4
KLHL35	Kelch-like family member 35
LKL	Kallikrein
LncRNA	Long non-coding ribonucleic acid
LRP1	Lipoprotein receptor-related protein 1
MAPK	Mitogen-activated protein kinase
MBD	Methyl-cytosine binding
Mcl-1	Myeloid cell leukemia 1
Mdm2	Mouse 3T3 cell double minute 2
mIR	Micro ribonucleic acid
miRNA	Micro ribonucleic acid
MMP	Matrix metallopeptidase
mRNA	Messenger ribonucleic acid
MRTF-B	Myocardin-related transcription factor-B
MYH11	Myosin heavy chain 11
NADPH	Reduced Nicotinamide adenine dinucleotide phosphate
NFκB	Nuclear factor kappa-light-chain-enhancer of activated B cells
Nkx2-5	NK-2 transcription factor
NOX	NADPH oxidase
Nt	Nucleotide
OPG	Osteoprotegerin
PCSK9	Propretein convertase subtilisin/kexin type 9
PDGF	Platelet-derived growth factor
PI3k	Phosphoinositide 3-kinase
PPARγ	Peroxisome proliferative-activated receptor gamma
PPE	Porcine pancreatic elastase
PTEN	Phosphatase and tensin homolog
PVT1	Plasmacytoma variant translocation 1
RECK	Reversion inducing cysteine-rich protein with kazal motifs
RIPK3	Receptor-interacting serine/threonine-protein kinase 3
RNA	Ribonucleic acid
ROS	Reactive oxygen species
SERPINA1	Serine peptidase inhibitor, clade A, member 1
Shh	Sonic hedgehog
shRNA	Short hairpin RNA
SMYD2	SET and MYND domain-containing 2
SNP	Single nucleotide polymorphism
SOD	Superoxide dismutase
SRF	Serum response factor
STAT	Signal transducer and activator of transcription proteins
TAA	Thoracic aortic aneurysm
TGFBR2	Transforming growth factor beta receptor 2
TGF-β	Transforming growth factor β
TIMP	Tissue inhibitor of metallopeptidase
TNFα	Tumor necrosis factor α
UCP	Uncoupling protein
UTR	Untranslated region
VSMC	Vascular smooth muscle cell
α1-AT	α1-antitrypsin
